# Development of a Statistical Model for Estimating Spatial and Temporal Ambient Ozone Patterns in the Sierra Nevada, California

**DOI:** 10.1100/tsw.2002.86

**Published:** 2002-01-17

**Authors:** Haiganoush K. Preisler, Michael J. Arbaugh, Andrzej Bytnerowicz, Susan L. Schilling

**Affiliations:** Pacific Southwest Research Station, 800 Buchanan St., West Annex, Albany, CA 94710, USA; Pacific Southwest Research Station, Forest Fire Laboratory, 4955 Canyon Crest Drive, Riverside, CA 92507, USA

**Keywords:** generalized additive models, jackknife estimates, passive samplers, random effects

## Abstract

Statistical approaches for modeling spatially and temporally explicit data are discussed for 79 passive sampler sites and 9 active monitors distributed across the Sierra Nevada, California. A generalized additive regression model was used to estimate spatial patterns and relationships between predicted ozone exposure and explanatory variables, and to predict exposure at nonmonitored sites. The fitted model was also used to estimate probability maps for season average ozone levels exceeding critical (or subcritical) levels in the Sierra Nevada region. The explanatory variables — elevation, maximum daily temperature, and precipitation and ozone level at closest active monitor — were significant in the model. There was also a significant mostly east-west spatial trend. The between-site variability had the same magnitude as the error variability. This seems to indicate that there still exist important site features not captured by the variables used in the analysis and that may improve the accuracy of the predictive model in future studies. The fitted model using robust techniques had an overall R2 value of 0.58. The mean standard deviation for a predicted value was 6.68 ppb.

## INTRODUCTION

Ozone injury to ponderosa and Jeffrey pines was first reported in the Sierra Nevada in the 1970s[1], and the spatial extent of injury was subsequently examined in national forests and parks using surveys[2,3]. Peterson et al.[4] evaluated crown conditions and derived basal area growth trends from cores collected from ponderosa pines in seven federal administrative units in the Sierra Nevada. The study found no evidence of recent large-scale growth changes in ponderosa pine in the Sierra Nevada; however, the frequency of trees with recent declines of growth was higher in the southernmost units.

In 1990 to 1991 two regional programs were initiated, the Sierra Cooperative Ozone Impact Assessment Study (SCOIAS) and the Forest Ozone REsponse STudy (Project FOREST). The parallel projects were conducted at the same locations in the Sierra Nevada from 1991 to 1994. SCOIAS's principal activity was to monitor ambient ozone and meteorological variables at six Sierra Nevada sites[5]. The FOREST project was developed as a companion project to SCOIAS through an agreement between the Forest Service, Region 5, Air Resource Management, and the California Air Resources Board. Project FOREST monitored the condition of pines and ozone air quality at ten locations along a north-to-south transect in the Sierra Nevada from Lassen Volcanic National Park in the north to Sequoia National Park in the south.

In 1999 a regional survey of seasonal ambient ozone exposure patterns in the Sierra Nevada was conducted using the Project FOREST and SCOIAS studies. A network of passive samplers was located along elevation gradients adjacent to active monitoring stations currently operated by the California Air Resources Board and the National Park Service. Around each active monitoring station, a network of passive ozone monitors was established, resulting in a total of 93 passive monitors (79 used in this analysis) located throughout the Sierra Nevada, of which 9 were colocated with active ozone monitors.

This study is concerned with the estimation of a predictive model for generating maps of ozone exposure over the Sierra Nevada over time. The modeling was done by using the passive sampler data and incorporated a spatial component (latitude, longitude) simultaneously with weather and other explanatory variables in a regression framework. Maps of the probabilities (risks) of seasonal cumulative ozone values exceeding critical levels were also developed as an alternative to estimated ambient ozone patterns and as a means for displaying measures of uncertainties.

## METHODS

### Study Area and Data

Sites for passive monitors were selected at three general elevations along the north-to-south gradient of air pollution on the western side of the Sierra Nevada. Mid-elevation monitoring sites were located at or near stands of ponderosa or Jeffrey pines that were subsequently sampled using Forest Pest Management survey protocol (1500 to 1750 m)[2]. Monitoring sites were also located at lower elevations of 1000 to 1400 m and at high-elevation sites located along the mixed conifer-subalpine ecotone at 2000 to 2400 m (Fig. 1). All sites were located at least 200 m from frequently used roads, in open areas that had good vertical mixing of air. Nine passive monitor sites were colocated with active monitors that operated during the 1999 summer season.

A single passive ozone sampler containing two cellulose filters saturated with nitrite was installed at each site[6]. Ozone oxidizes nitrite into nitrate ions, and the amount of nitrate in the filter at a given time is a measure of the amount of ozone at the site. The samplers were located at about 1.5 to 2.5 m above ground level in forest clearings about 20 m or more from the nearest trees. At eight to ten monitoring sites in each 2-week collection period, two blank filters were also tested. Blank filters were kept at room temperature in tightly closed plastic vials. In the field, the filters were changed every 2 weeks during the summer growing season. After the exposures, the filters were placed in plastic vials and refrigerated until analyzed. Ozone concentrations were continuously monitored by UV absorption (Thermo Environmental Model 49, Cambridge, MA, or an equivalent instrument), at nine monitoring stations for comparison with colocated passive samplers.

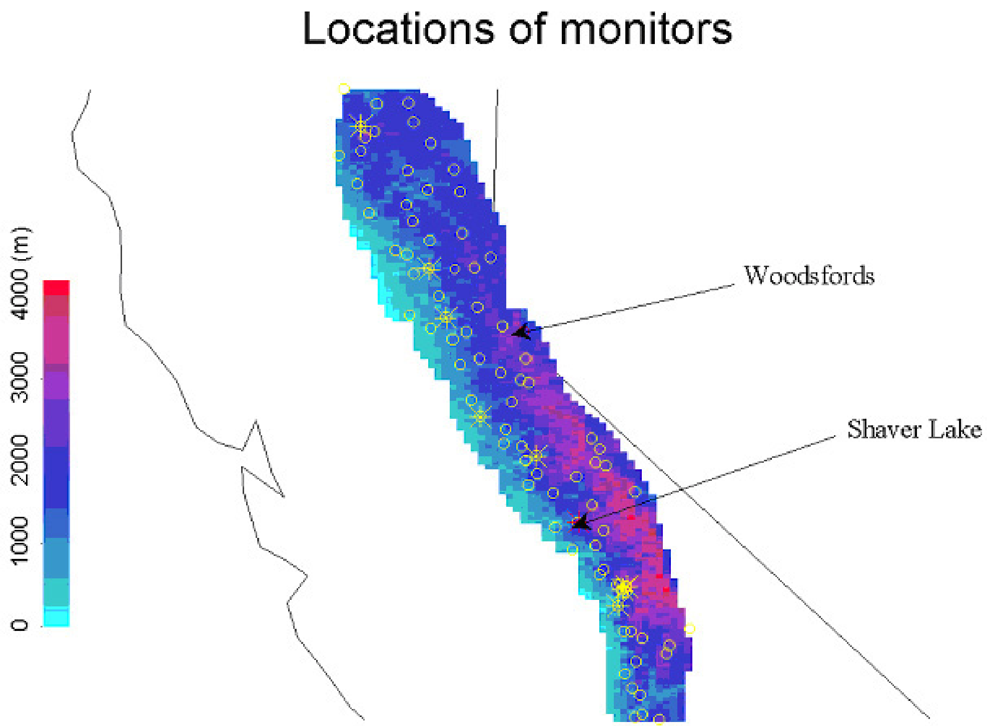

**FIGURE 1.** An elevation map and locations of passive and colocated active monitoring stations in the Sierra Nevada.

Daily maximum temperature and precipitation were obtained from 55 weather stations distributed over the Sierra Nevada range. Elevations at monitoring sites and at the weather stations were obtained from the National Climatic Data Center (NCDC) Summary of the Day Database (Earthinfo, 1992) and the National Interagency Fire Management Integrated Database (NIFMID) from the USDA National Information Technology Center.

## Statistical Methods

### *Estimating Relationship Between Ambient Ozone and Nitrate Oxidation Rates*

Passive samplers are based on ozone oxidizing nitrite ions, which are coated onto filters, into nitrate ions. The chemical reaction in the filters is

$$NO_2^- + O_3 \xrightarrow{\beta} NO_3^- + O_2$$

where β is the rate of the reaction. Consequently, the amount of $NO_3^-$ in a filter at a given time is

$$NO_3^- = \alpha + \beta \times O_3$$

where α and β are unknown parameters to be estimated from colocated passive and active samplers. Given estimates for α and β, the formula $O_3 = (NO_3^- - \hat{\alpha}) / \hat{\beta}$ may be used to convert observed nitrate values to ozone levels at a given site.

The values of $\alpha$ and $\beta$ were estimated by regressing nitrate levels against ozone levels from colocated sites. Regression techniques were also used to study the effects of three variables (elevation, maximum temperature, and precipitation) on the values of the conversion factors, $\alpha$ and $\beta$. Depending on the results of the regression analyses, nitrate to ozone conversion may be done in one of three possible ways:

1. One common $\alpha$ and $\beta$, from all colocated sites is used in the conversion.
2. A separate $\alpha$ and $\beta$, for each colocated site is used. At sites with no colocated active monitors values from the nearest active site are used.
3. The estimated relationships between conversion factors and the covariates (elevation, maximum temperature, precipitation) are used to convert nitrate to ozone at sites with no colocated active monitors.

## A Spatial Temporal Regression Model for Estimating Ozone Maps

Modern regression techniques[7] were used to estimate ozone patterns for the Sierra Nevada region at various dates, given data from the network of passive samplers described above. The regression techniques employed in this analysis use the correlations between explanatory variables (e.g., weather and topographic variables) and ozone levels, plus spatial and/or temporal trends in ozone levels (i.e., correlations in space and time), to aid in predicting ozone levels at unobserved sites or dates. The techniques generalize classical multiple regression methods in that no linear or quadratic assumptions are made about the relationships between the dependent and the independent variables in the regression equation. Instead, by using scatter-plot smoothing tools (e.g., smoothing splines, kernel or near-neighbor smoothers, or locally weighted polynomials) it allows various terms in the regression equation to be added nonparametrically. Consequently, in modern regression techniques the data suggest the nonlinearities that might exist between the variables. This capability is most useful when fitting spatial data because spatial patterns are usually too complicated to be modeled by a simple polynomial function. Fitting polynomial trend surfaces is particularly problematic when observations are not regularly spaced because ordinary regression gives equal weights to each observation. Consequently, densely sampled regions may have larger effects on the estimates. Alternatively, modern regression techniques use locally weighted polynomial regressions[8] by fitting a separate linear or quadratic curve at each observation point using regression with weight functions, such as the tri-cube function, centered at the point. One of the arguments of locally weighted regression routines is the span, which controls the amount of smoothing. Using a large span results in smoother but less local fitted functions, whereas using a small span results in more local fitting (rougher fits). The particular regression model used to analyze the passive sampler data is described next.

Let $Y_{ijk}$ be the amount of nitrate in the filter of the $k^{th}$ sample (replicate), at the $i^{th}$ site, and day $t_{ij}$. We used a locally weighted regression model with random effects to characterize the relationship between the amounts $Y_{ijk}$ and the explanatory variables. Specifically, we used the model

$$Y_{ijk} = \alpha + g_0(lon_i, lat_i, t_{ij}) + \sum_l g_l(X_{lij}) + \tau_i + \varepsilon_{ijk} \qquad (1)$$

where $X_{lij}$ = value of the $l^{th}$ explanatory variable (e.g., temperature or elevation), $lon_i$,$lat_i$ = longitude and latitude of the $i^{th}$ site, the location of the $i^{th}$ passive sampler, $\alpha$ = overall mean nitrate level, $\tau_i$ = unobserved random site effect assumed to be Gaussian with mean zero and variance $\sigma_\tau^2$, $\varepsilon_{ijk}$ = unobserved independent random noise with mean zero and variance $\sigma_\varepsilon^2$, and

$g(.)$ = nonparametric functions estimated simultaneously by using a scatter-plot smoother iteratively.

Four explanatory variables ($l$ = 1,…,4) — maximum temperature, precipitation, ambient ozone level at nearest active site, and elevation — were used in the model. The first three variables were spatially and temporally explicit (i.e., had different values at different locations and times) while the fourth variable (elevation) was spatially explicit. Ozone level at nearest active site was used as an explanatory variable because, just as the other auxiliary variables such as temperature, it might be a useful predictor of nitrate levels at nearby sites without monitors. The smooth function of location and time, $g_0(lon_i,lat_i,t_{ij})$, was included in the regression to account for general spatial patterns not explained by any of the four covariates (e.g., patterns due to wind that may be added to the model if data on wind were available). A random site effect was included in the model to account for site-specific characteristics due to unknown or unobserved site covariates such as aspect. The between-record error terms, $\varepsilon$, were assumed to be independent. Similarities between observations at nearby sites were partially accounted for by including the smooth function of location in the equation of expected values. Directional variograms of the residuals were produced to check if any autocorrelations still remain. If autocorrelation is detected in the residuals, even after removing the effects of general spatial trends or spatially explicit covariates, then kriging techniques may be used on the residuals to obtain better predictions at unobserved sites[9].

Estimation of the nonparametric functions in Eq. 1 was done by the *gam* procedures in S-Plus[10,11]. However, the standard versions of these programs do not include allowances for the presence of random effects such as the random site effect assumed in the model (Eq. 1). To estimate the random effects component of the model, an iterative procedure was used based on the expectation-maximization (EM) algorithm[12] and the equations given in Brillinger and Preisler[13]. The EM algorithm involves the successive maximization of the expectation of the 'complete data' likelihood, conditional on the observed data. In our case the 'complete data' is the observed data and the current estimates of the random effects terms.

The weather variables — maximum temperature and precipitation—at the passive sampler sites were estimated from values recorded at 55 weather stations distributed over the Sierra Nevada. The model used to estimate weather data at the monitor sites, or any other location with no local weather station, was similar to the regression model in Eq. 1, but with the dependent variable replaced by maximum temperature (or precipitation) and with latitude, longitude, time in days, and elevation as explanatory variables.

## Standard Error Estimation

A modification of the grouped jackknife procedure[14] was used to estimate standard errors of the predicted values. The grouped jackknife procedure is based on leaving a subset of observations out at a time, then running the estimation procedure on the remaining observations. The standard errors of the predicted estimates are then calculated from the pseudo-values $\hat{\mu}_{\mathrm{k}} = k\hat{\mu}\text{-}(k\text{-}1)\hat{\mu}_{(\mathrm{k})}$ where $\hat{\mu}$ is an estimate using all the data and $\hat{\mu}_{(\mathrm{k})}$ is an estimate with the $k^{th}$ group removed. A natural grouping of the nitrite oxidation rates was the groups of observations from each site. However, the variability in the pseudo-values obtained by dropping one site at a time was very large. Some of the sites had a large influence on the estimated values, with the effect that standard error estimates calculated in this fashion were too large. This was verified with a simulation study in which we were able to compare the estimated jackknife standard errors with the true standard errors. The standard error calculations were made using a modified jackknife procedure in which the observations from deleted sites were replaced by their expected values estimated from the rest of the data[15].

### Assessing Goodness of Fit of Model

Normal probability plots of the residuals were produced to assess the Gaussian assumption of the model in Eq. 1. Cross-validation techniques were used to produce plots of observed vs. expected values, with expected values at a given site calculated using data from all other sites. Another useful set of plots for assessing overall goodness of fit were those of the estimated ozone values compared with the observed ozone values at the colocated sites. Estimated directional variograms of residuals plotted against distance were used to assess the assumption of spatial independence of the error terms.

### Formulas for Estimating Probability Maps for Season Average Ozone Levels

Maps that show spatial patterns of ozone levels do not include measure of uncertainties of the estimates. A second map is usually needed to show the standard error estimates of predicted values. One way of including predictions and uncertainties in the same map is to show the estimated probabilities (or risks) of ozone values that exceed certain critical levels. Such maps are especially useful for end-of-season measures, such as the average ozone level for the season at a given location. In this study the season was a 10-week period starting May 25. Using the model in Eq. 1, the probability of the average over a given period of time exceeding a critical amount is given by

$$\Pr[Sav_i > C] = 1 - \Phi\left(\frac{C - M_i}{\sqrt{\mathrm{var}(Sav_i)}}\right) \qquad (2)$$

where $Sav_i$ = average ozone level for site $i$ over a particular period of $N$ days, $C$ = a critical concentration of ozone (e.g., 60 ppb), $\Phi$ = standard Gaussian distribution function, $M_i = \hat{\mathrm{E}}(Sav_i) = \frac{1}{J}\sum_{j=1}^{J}\hat{\mu}_{ij}$ , $\hat{\mu}_{ij}$ = estimated amount of hourly ambient ozone level at site $i$ and for the $j^{th}$ 2-week collection period, and $J = N/14$ and var($Sav_i$) = variance of the season average ozone level at site $i$.

An estimate of the variance of $Sav$ for the random effect model used in this study is given by the formula

$$\hat{\mathrm{var}}(Sav_i) = \frac{1}{J}[\hat{\mathrm{var}}(\hat{\mu}_{ij}) + \hat{\sigma}_{\varepsilon}^2 + J\hat{\sigma}_{\tau}^2]$$

## RESULTS

Maps of estimated hourly ambient ozone levels for two dates in 1999 are produced in Fig. 2. The patterns seen on those dates were typical of the general pattern of ozone levels during summer 1999. Namely, ambient ozone levels in 1999 appeared to be highest in some of the western and eastern regions of the southern Sierra Nevada. This result is consistent with previous reports of observed foliar injury[5,16]. Maps of estimated probabilities for average ozone levels over a period of 140 days starting May 25 are produced in Fig. 3. These maps indicate with 95% certainty that all of the Sierra Nevada was exposed to average seasonal levels of 40 ppb or greater. Only the central and southwestern Sierra Nevada were likely to have been exposed to average seasonal values greater than 60 ppb. One southeastern area of the Sierra Nevada also appeared to have been exposed to high levels of ozone (>60 ppb) with 95% certainty.

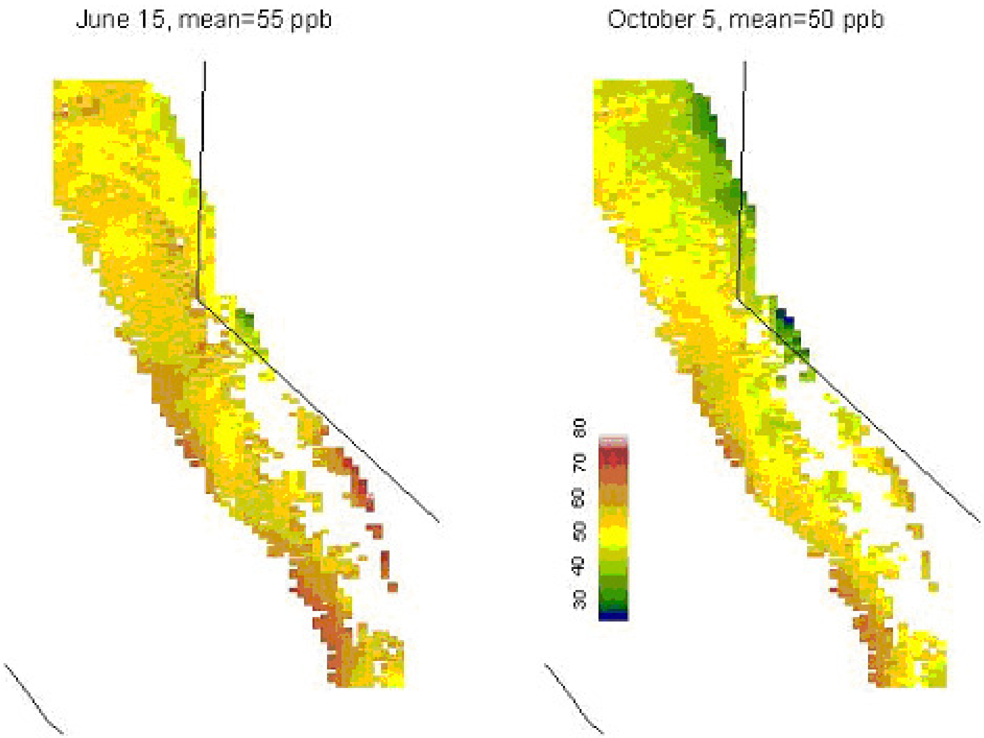

**FIGURE 2.** Estimated ambient ozone levels for the Sierra Nevada region on 2 days during summer 1999. Estimates of ozone at the highest elevation sites (the regions in white) are not available because there were no passive sampler monitors at elevations >2500 m.

The relationships between ambient ozone levels and observed nitrate levels at colocated passive and active monitor sites are presented in Fig. 4. The slopes and intercepts of the regression lines in Fig. 4 were significantly different for the various sites. The nitrite oxidation–rate relationship with the continuous monitor at Shaver Lake site (see Fig. 1) was extreme relative to relationships at other continuous monitor sites. Examination of the data indicated that although nitrite oxidation rates were comparable with sites north and south of Shaver Lake, continuous monitor values were almost 50% lower than nearby continuous monitors. The Shaver Lake continuous monitoring station was accordingly excluded from further analysis. One of the measured site characteristics (elevation) appeared to have a significant effect on the slopes and intercepts of the regression lines. There is some indication that slope was lower than average and the intercept is higher than average at elevations less than 1000 m (Fig. 5). The relationships between the slopes and intercepts and the measured weather variables (maximum temperature and precipitation) were found to be not significant ($p$-values > 0.8). Although this seems to indicate that nitrate to ozone conversion factors should be based on some local site conditions, additional data are needed before this method can be used. Also, using a separate slope and intercept for each site did not produce any discernable effect on the final maps that described estimated spatial ozone patterns. Consequently, a common slope and intercept was used in this study to convert nitrate levels to ozone levels at all sites.

There were 24 values in the normal probability plot that appeared to be smaller or larger than expected under the assumed model (see Fig. 6). These values may indicate either the need for more accurate or additional explanatory variables. For example, the maximum temperature values used at each site were the values of the smoothed surface of temperatures estimated using weather

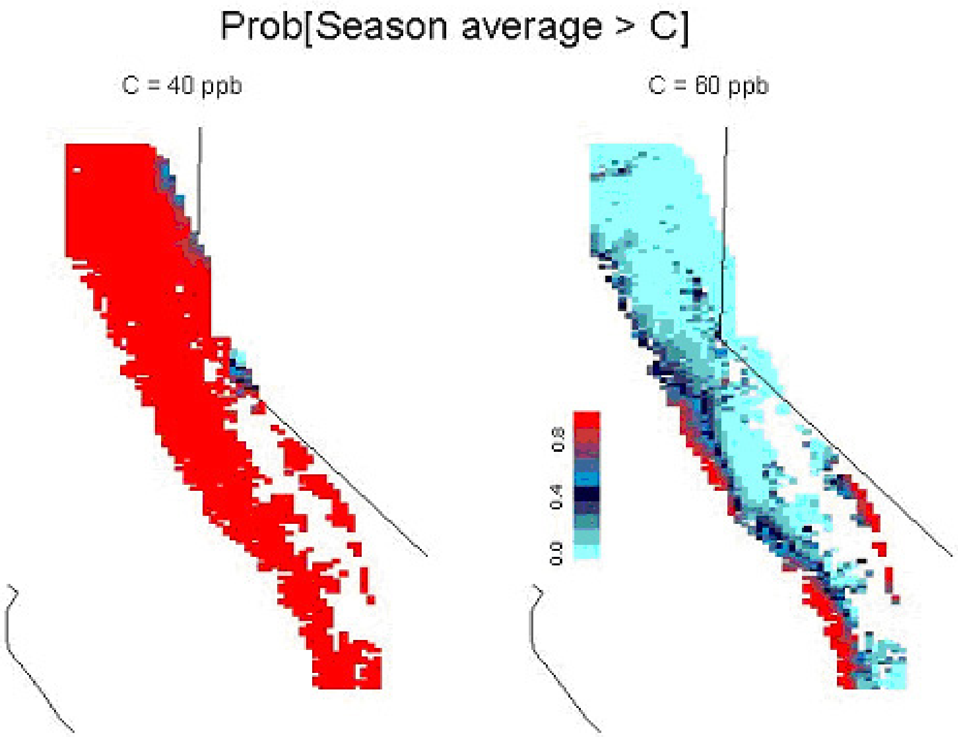

**FIGURE 3.** Estimated probabilities of seasonal average ozone levels exceeding 40 and 60 ppb. The season was a period of 140 days starting May 25, 1999.

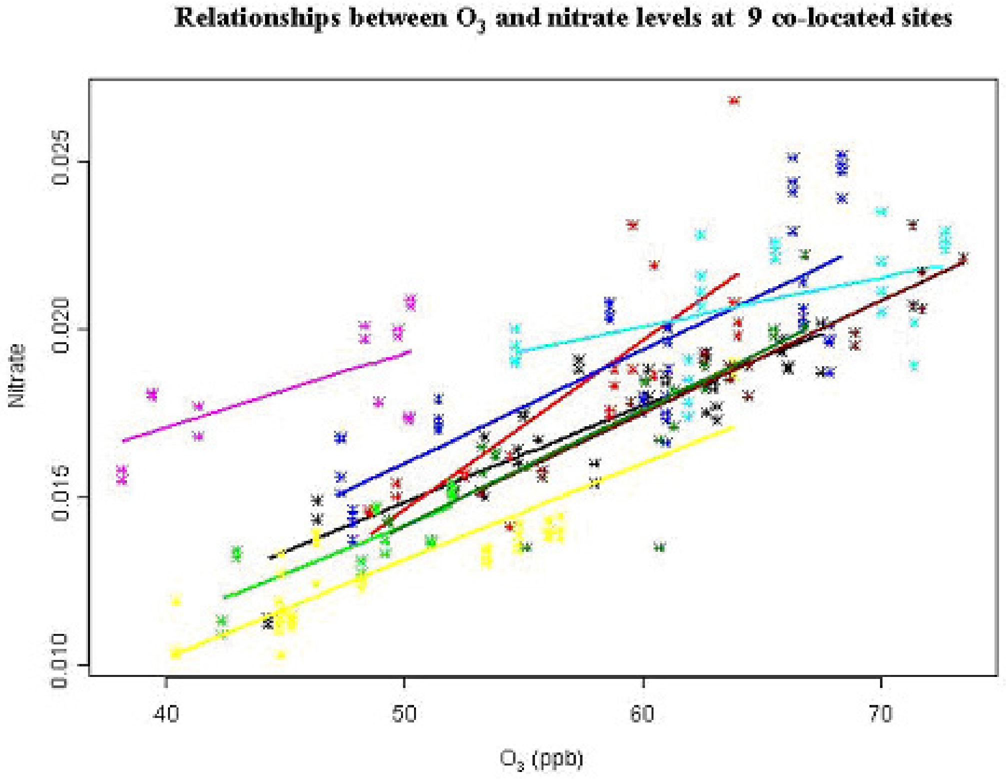

**FIGURE 4.** Relationships between ozone levels observed at active monitors and those observed by passive samplers at the colocated sites. The active-monitor data from one of the sites (Shaver Lake) appears to be an outlier.

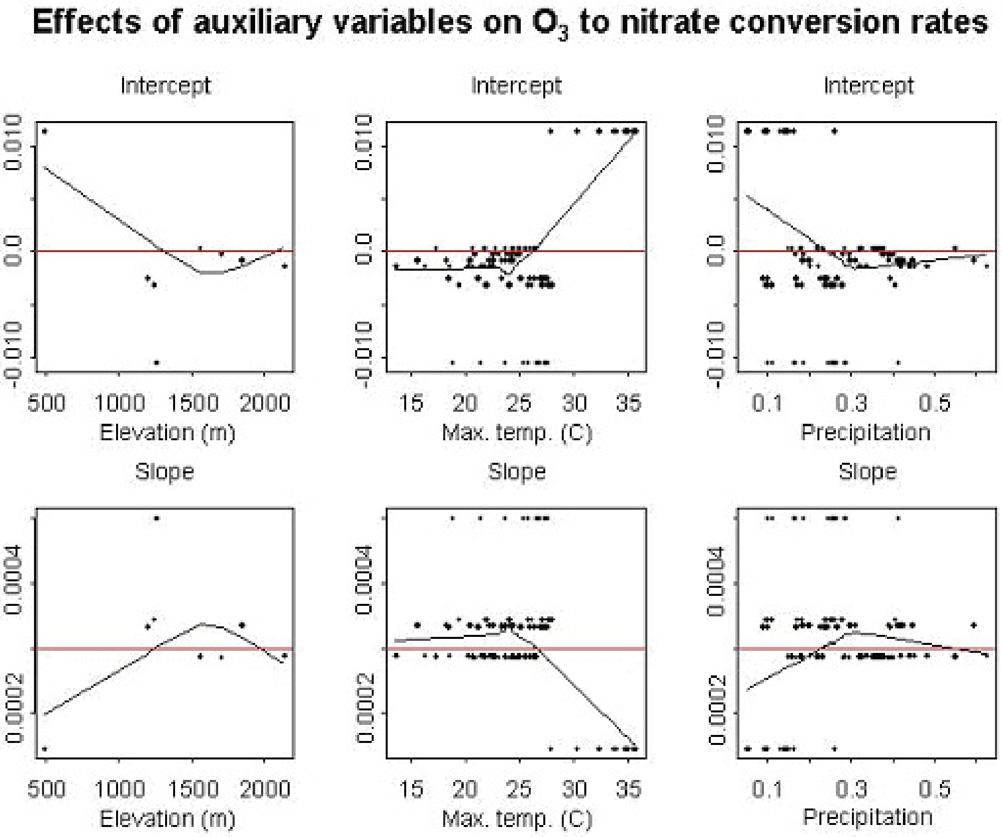

**FIGURE 5.** Relationships between auxiliary variables and the slopes and intercepts of the $O_3$-to-nitrate conversion relationships. The horizontal lines indicate the average levels. Only the relationships with elevation are statistically significant.

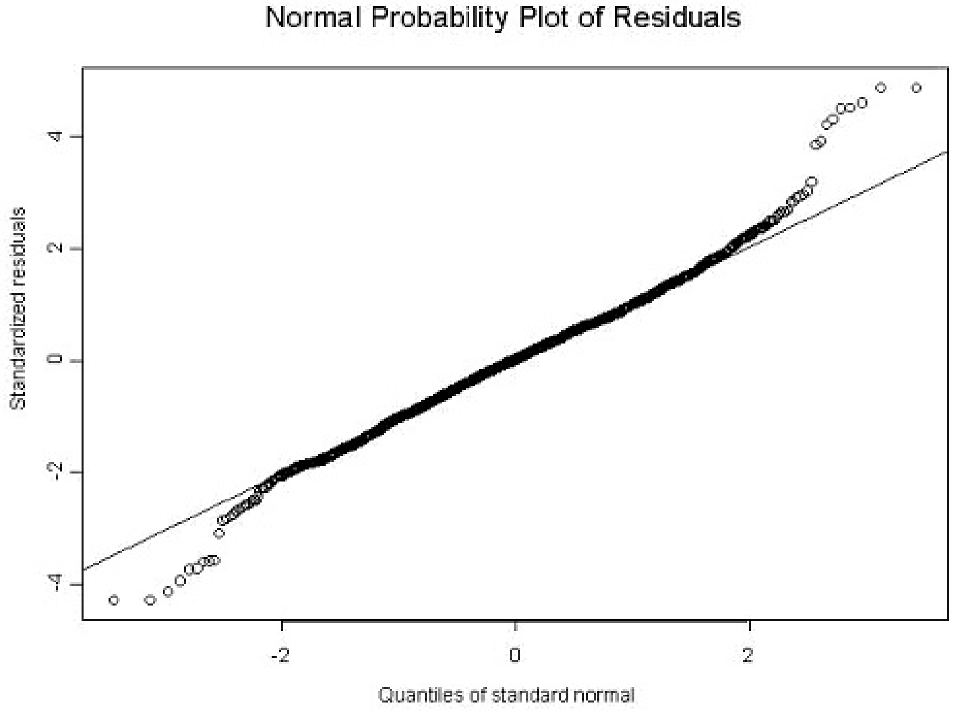

**FIGURE 6.** Normal probability plot of the residuals after fitting the model to the nitrate data observed at 79 passive monitors over 5 months.

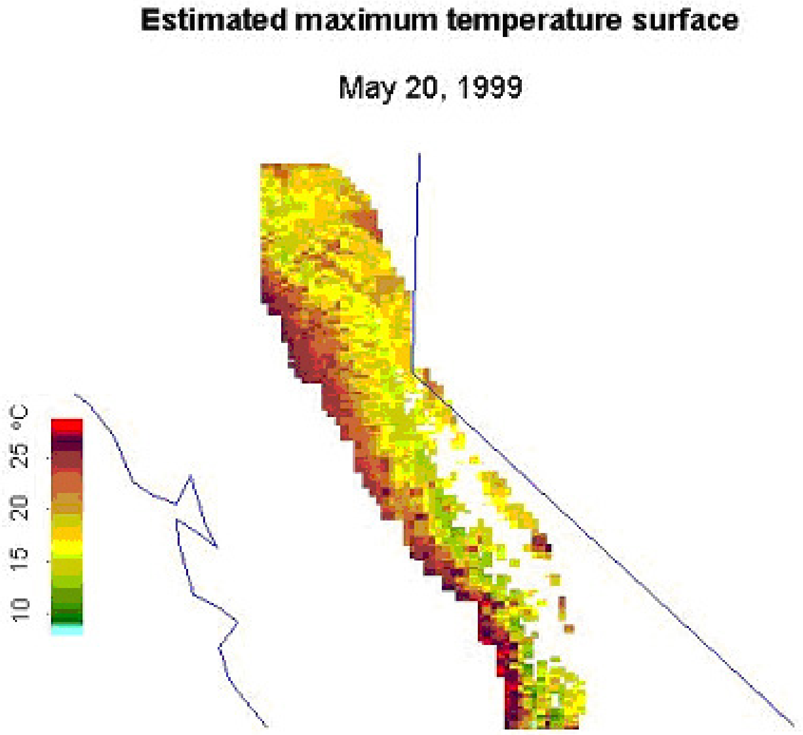

**FIGURE 7.** Maximum temperature surface for 1 day of the season estimated from data recorded at 55 weather stations scattered throughout the Sierra Nevada. The smooth surface was generated using locally weighted regression with location, elevation, and day as covariates.

station data, as shown in Fig. 7. Site-specific temperatures produced in this fashion are usually not good estimates of local extreme values. Although this might not justify the additional expense of locating meteorological stations with each passive monitor, nevertheless it indicates the need for further studies at a finer scale, possibly using data from a few sites where weather stations and passive sampler monitors are colocated.

Plots of estimated directional variograms of model residuals, shown in Fig. 8, indicated that the assumption of spatial independence of the error terms was adequate. The variograms were basically flat, indicating no autocorrelation. Approximately 94% of the observed ozone values were within the estimated point-wise 95% confidence bounds produced by the cross-validation study (see Fig. 9). Some of the points outside the 95% bands were the extreme values already discussed above. However, a new group of outliers (all from Woodsfords site in Eldorado Forest, see Fig. 1) were detected. All the observed values at this site were greater than two standard deviations from the expected values.

Estimated between-site variation ($\hat{\sigma}_\tau$ = 3.8 ppb) was significant when compared with the record-to-record variation ($\hat{\sigma}_\varepsilon$ = 5.4 ppb). Approximately 33% of the total variation was due to between-site variations. This effect is also evident in Fig. 10, in which observed and fitted values of ambient ozone levels at nine sites are compared. The Woodsfords site was the outlier site mentioned above. None of the auxiliary variables included in the model appear to account for the larger than expected ozone levels at this site. Also, the increasing trend seen at one of the Sierra National Forest (NF) sites in Fig. 10 does not seem to be accounted for with the temporally explicit weather covariates in the model. Although similar increasing trends at other sites (e.g., the Sequoia NF site) seem to be well-accounted-for by the weather covariates. The relatively large between-site variation, and the bias seen at some of the sites in the predicted values as a

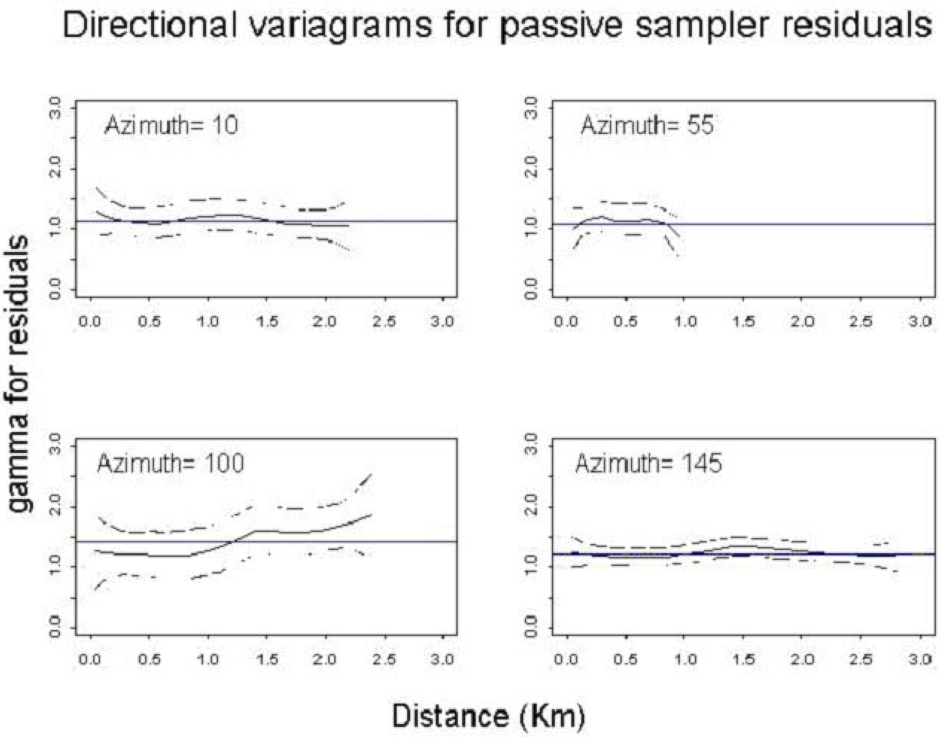

**FIGURE 8.** Estimated directional variograms (and 95% confidence limits) of the residuals. The variograms are mostly flat, indicating that the fitted model has accounted for most of the spatial autocorrelations in the data. The four panels refer to spatial autocorrelation patterns in four azimuth directions from passive sampler sites.

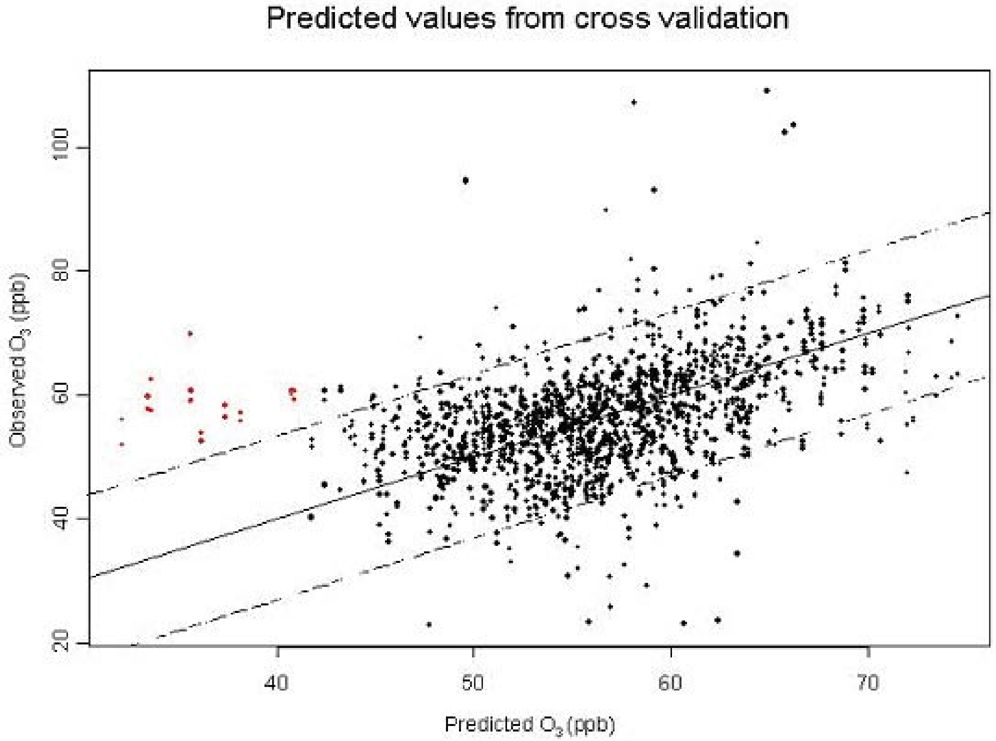

**FIGURE 9.** Observed vs. predicted values from a cross-validation study where values at a site are predicted using data from the remaining sites. According to the model, 95% of ozone levels are expected to fall within the dashed lines. Predicted values for one of the sites in Eldorado Forest (red dots) appear to be biased.

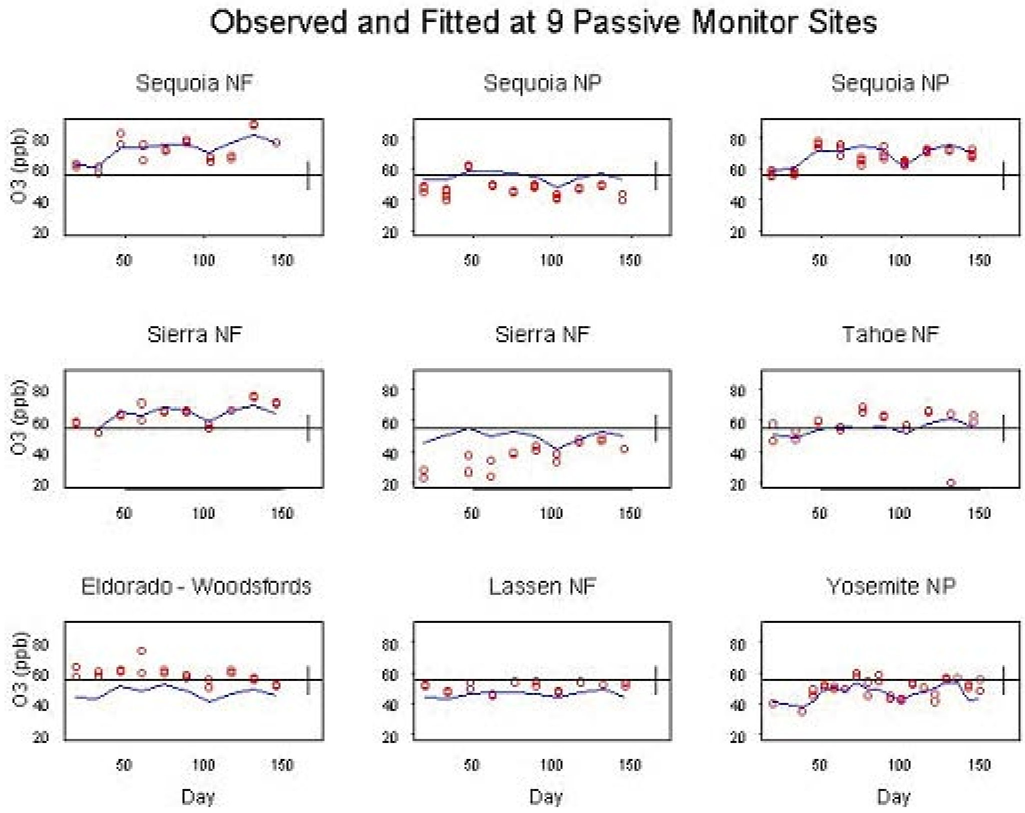

**FIGURE 10.** Observed vs. estimated ozone levels at nine sites. The horizontal line is the seasonal average ozone level. The length of the vertical bar on the right side of each panel is twice the maximum predictive standard error estimated using the jackknife technique.

consequence (e.g., the Eldorado and Lassen NF sites in Fig. 10) seem to imply that the model prediction could be improved if additional site characteristics (e.g., aspect) are included in the model. Another explanation for the relatively large between-site variations might be the need for better *in situ* calibration of the monitors.

The conditional effects of each of the explanatory variables (including spatial location), after controlling for the effects of the other variables shown in Fig. 11, indicate that although all six variables were significant at the 5% level, maximum temperature and elevation had the largest effect on ozone levels. The explanatory variables were not independent, hence relationships may differ from those obtained from single-variable analysis. For example, part of the effect of elevation commonly observed may be due to lower temperature values at higher elevation. Since temperature is one of the variables in the model, the elevation effect seen in Fig. 11 is likely to be a proxy for some other variables that not included in the model but are correlated with elevation, e.g., higher levels of radiation at higher elevation. The spatial effect included in the model is likely to be a proxy for other spatially explicit variables such as titration of ozone by NO at lower elevation locations closer to pollution source areas.

## CONCLUSIONS

The development of statistical models describing patterns of ambient ozone over space and time is now practical due to the development of low-cost passive sampler systems. In this initial modeling study a spatial-temporal model was developed with an estimated $R^2$ = 58% and with an average standard deviation of 6.68 ppb. Further improvements in the accuracy of predictions are likely, by including additional explanatory variables.

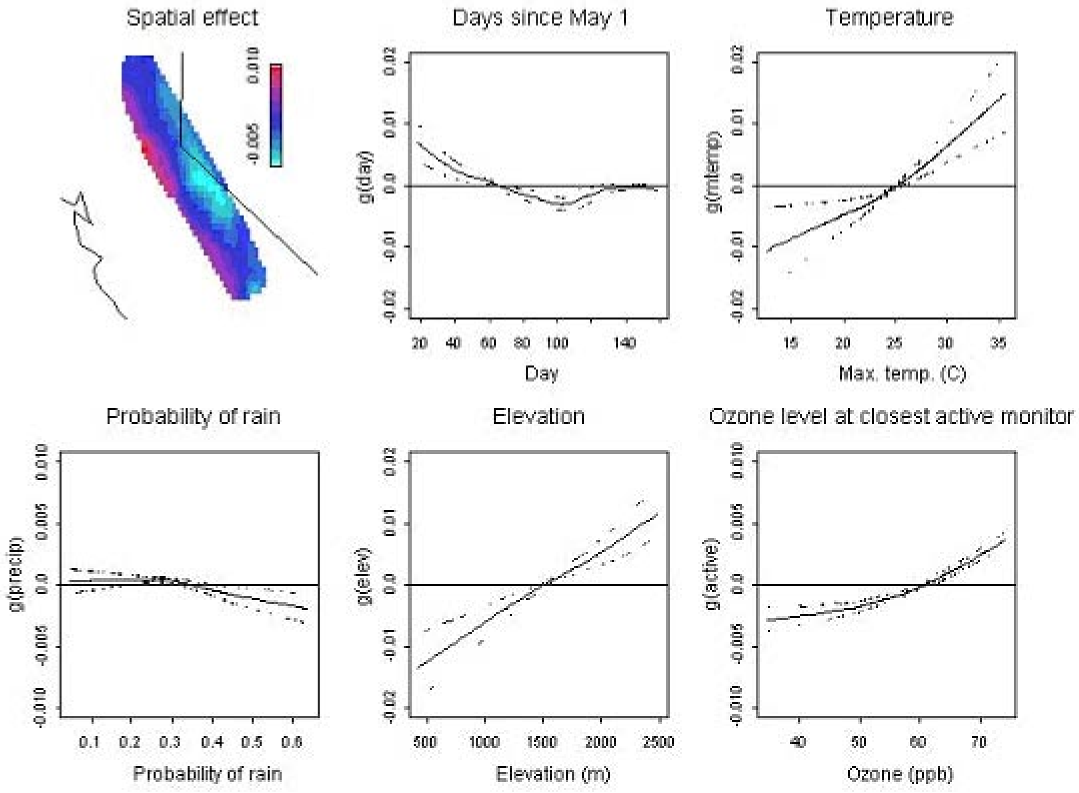

**FIGURE 11.** Estimated conditional effects (and 95% jackknife confidence bands) of the explanatory variables. Days since May 1 indicates the cumulative number of days from the beginning of the study.

This study illustrates that spatial maps of ambient ozone exposure, and possibly effects on vegetation, can now be developed for large regional wildland areas. This information should aid air quality resource managers in assessing the risk of adverse air quality effects on wildland and on human communities.

The statistical approach used in this study was an extension of ordinary linear regression techniques to nonlinear and spatially correlated cases. Defining the model as a regression model provided a flexible framework for determining uncertainties, assessing goodness of fit, and detecting observations that are not adequately predicted by the model. The latter should aid scientists in finding ways to improve the model, such as by identifying additional explanatory variables.

## ACKNOWLEDGEMENTS

We would like to thank Rocio Alonso for providing us with ambient ozone data from the active monitors and Nancy Grulke for discussion on cumulative ozone levels and passive sampler calibration.
